# Porous Iron Electrodes Reduce Energy Consumption During Electrocoagulation of a Virus Surrogate: Insights into Performance Enhancements Using Three-Dimensional Neutron Computed Tomography

**DOI:** 10.1021/acsestengg.4c00317

**Published:** 2024-09-23

**Authors:** Kyungho Kim, Cesar Castillo, Gyoung G. Jang, Yuxuan Zhang, Costas Tsouris, Shankararaman Chellam

**Affiliations:** † Department of Civil & Environmental Engineering, Texas A&M University, College Station, Texas 77843, United States; ‡ Manufacturing Science Division, Oak Ridge National Laboratory (ORNL), Oak Ridge, Tennessee 37831, United States; § Neutron Scattering Division, ORNL, Oak Ridge, Tennessee 37831, United States

**Keywords:** electrified treatment, porous electrode, microbial control, neutron computed tomography, energy saving

## Abstract

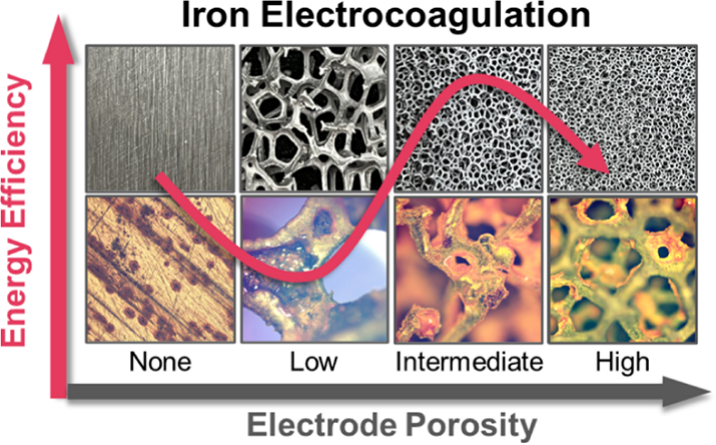

Electrocoagulation has attracted significant attention as an alternative to conventional chemical coagulation because it is capable of removing a wide range of contaminants and has several potential advantages. In contrast to most electrocoagulation research that has been performed with nonporous electrodes, in this study, we demonstrate energy-efficient iron electrocoagulation using porous electrodes. In batch operation, investigation of the external pore structures through optical microscopy suggested that a low porosity electrode with sparse connection between pores may lead to mechanical failure of the pore network during electrolysis, whereas a high porosity electrode is vulnerable to pore clogging. Electrodes with intermediate porosity, instead, only suffered a moderate surface deposition, leading to electrical energy savings of 21% and 36% in terms of electrocoagulant delivery and unit log virus reduction, respectively. Neutron computed tomography revealed the critical role of electrode porosity in utilizing the electrode’s internal surface for electrodissolution and effective delivery of electrocoagulant to the bulk. Energy savings of up to 88% in short-term operation were obtained with porous electrodes in a continuous flow-through system. Further investigation on the impact of current density and porosity in long-term operation is desired as well as the capital cost of porous electrodes.

## 1 Introduction

Access to clean and safe drinking water is a fundamental requirement for the well-being and health of populations worldwide.^1^ However, the escalating challenges posed by pollution and scarcity have urged researchers to explore innovative and sustainable solutions for water treatment.^2,3^ Electrified technologies such as electrooxidation (EO), electrocoagulation (EC), and electrosorption harness electricity to drive electrochemical reactions to combat target pollutants. They are capable of treating a wide range of pollutants, while simultaneously reducing costs and safety concerns associated with transporting and storing of chemicals needed for conventional treatment technologies, and even potentially reducing energy consumption.^4,5^

EC has emerged as a promising technology to replace traditional chemical coagulation. Extensive studies on EC application for domestic and industrial wastewater treatment have shown that EC is capable of removing a variety of contaminants including suspended solids, metals, microorganisms, toxic organic/inorganic compounds, etc.^6−8^ EC has also been proven to accomplish more than any separation technology alone can achieve. Successful mitigation of membrane fouling, as well as improving the water quality when employed as a pretreatment of membrane filtration processes demonstrates the synergistic role of EC as a treatment train component.^7^ Importantly, EC is also capable of chemically transforming contaminants by generating strong oxidants such as reactive chlorine/oxygen species and ferryl iron enabling it to degrade organic substances.^8−11^ This has initiated a growing interest in hybridizing EC with other advanced processes such as electro-Fenton, EO, and ozonation.^8,9^ Hence, EC is potentially more versatile than conventional chemical coagulation as a water treatment technology. In order for an electrochemical process to be competitive against a mature conventional counterpart and a feasible option, its operational cost has to be relatively low.^12^ In an effort to reduce the electrical energy needed to drive electrochemical reactions, a main component of system expenses, porous electrodes have been employed for numerous electrified processes taking advantage of a large specific surface area allowing for the utilization of both the inner and outer surfaces, thereby lowering cell voltage.^13,14^ However, the application of porous electrodes has been scarcely explored for EC. A single study is available wherein energy-saving EC with porous aluminum electrodes was demonstrated in comparison with EC utilizing solid electrodes.^15^

In this context, this study aims to contribute to the body of knowledge on EC as an advanced water treatment technology by evaluating the performance of porous iron electrodes in terms of energy efficiency and water quality enhancement. Iron foams with various porosities were explored in comparison with a solid iron plate for electrical energy consumption and virus control in a synthetic secondary effluent, an important nontraditional water source. Further, 3-dimensional neutron computed tomography (nCT) was employed as a nondestructive tool to investigate changes in internal pore structures focusing on revealing the involvement of internal pore surfaces in anodic dissolution, which is a critical factor for porous electrode performance. nCT has the ability to provide detailed internal and external images of a sample structure based on a planar or volumetric map of neutron attenuation coefficients.^16−19^ In contrast to X-ray, γ-ray, and transmitted electrons that tend to preferentially interact with heavy elements, neutrons penetrate dense elements such as iron and strongly interact with light elements generating high contrast between elements by orders of magnitude.^20−22^ Therefore, nCT was considered to be a suitable tool for iron electrodes for which changes including deposition of light elements such as H, O, C, Ca, Si, etc. were expected.^23,24^ To the best of our knowledge, this is the first study to apply porous iron electrodes for EC.

## 2 Materials and Methods

### 2.1 Solid and Porous Iron Electrodes

EC experiments were performed with both solid and porous electrodes. For a solid electrode configuration, two identical low-carbon (0.2%) iron plates were employed as both anode and cathode (McMaster-Carr, 99% Fe, 4.8 cm width × 0.3 cm thickness). For a porous electrode configuration, a pair of identical iron foam sheets (Stanford Advanced Materials, ≥95% Fe) was used as an anode and a cathode. To investigate the effect of pore size, the porosity of iron foam was varied as 10, 50, and 100 pores per inch (PPI) while the width and thickness of each electrode were kept the same as those of the solid electrodes. Table 1 presents the images of the plate-type solid and porous electrodes and structural properties such as the ratio of electrode surface area to electrode bulk volume and porosity.

**Table 1:**
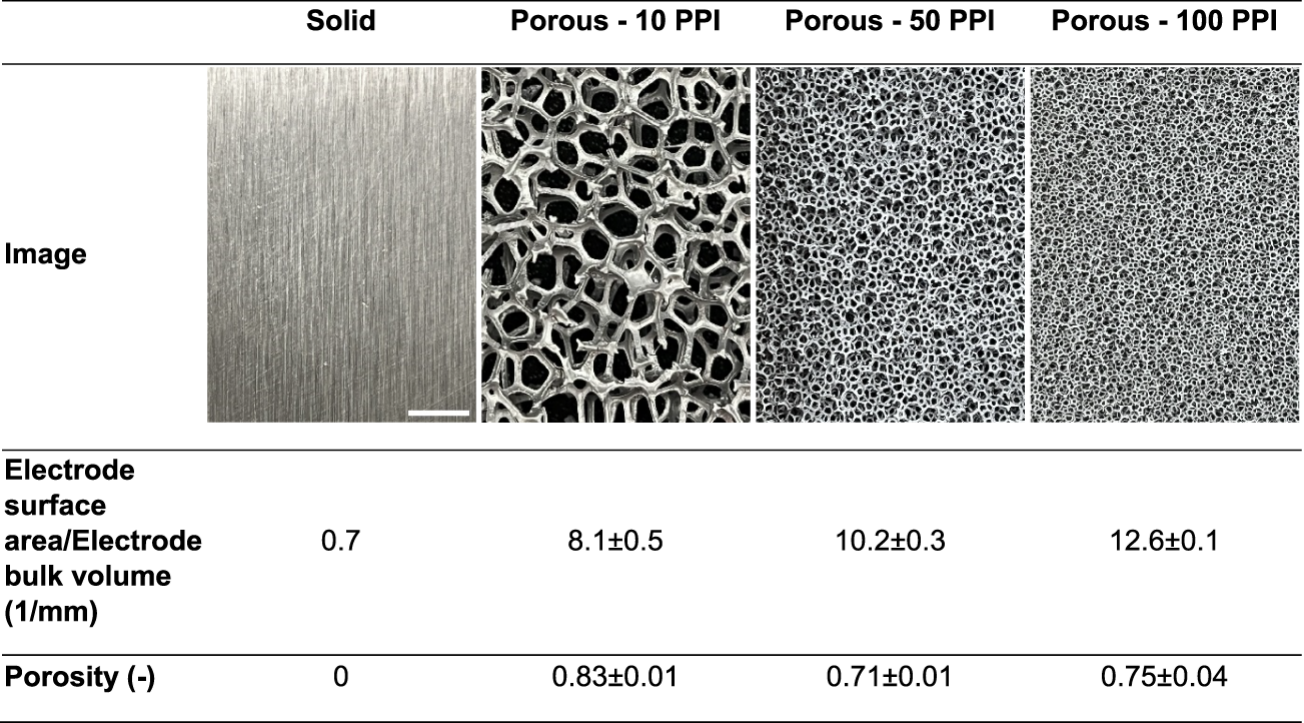
Photos of Pristine Solid Iron Plate (Left) and Porous Iron Foam Electrodes of Varying Porosity (Expressed in Pores Per Inch, i.e., PPI) Used in This Study^a^

a The scale bar in the far-left panel represents 5 mm and applies to all the other panels. Structural properties of electrode surface area/electrode bulk volume and porosity are also presented based on the tomography results.

### 2.2 Batch EC Reactor and Operation

A batch reactor was fabricated using a borosilicate glass beaker (8.5 cm inner diameter × 11.5 cm height) equipped with slim rectangular baffles (8.5 cm height × 1.5 cm width × 0.1 cm thickness) on the wall and a rod-shaped magnetic stirrer (5 cm length × 0.8 cm thickness) for mixing. Based on the two actual cases of secondary effluent potable reuse^25−27^ shown in the Table S1, a synthetic solution was freshly prepared before each experiment. It is noted that the synthetic solution closely matched the inorganic composition of the two real-world cases but was organics-free for sake of simplicity (i.e., to focus on comparing solid and porous electrodes and investigating the impact of inorganic elements). The reactor was filled with 500 mL of synthetic secondary effluent and then, a pair of solid or porous electrodes were immersed at 5 cm deep having a 1 cm gap between them, providing a submerged area of 52.4 cm^2^ for each electrode. Note that this area does not represent the actual surface area of the porous electrodes exposed to the solution. Electrolysis was immediately conducted at a constant current of 0.05 A (i.e., 0.95 mA/cm^2^ for the solid electrode) for 11.5 min using a software-controlled potentiostat (Interface 1010E, Gamry Instrument) with a vigorous mixing aiming to add 20 mg/L total Fe in the bulk water. The calculation of electrolysis time is shown in Section S2. The electrodes were removed from the suspension after the electrolysis to eliminate the chemical dissolution of the electrodes due to dissolved oxygen and high ionic component concentrations.^28^ The current and cell voltage were automatically recorded by the software.

### 2.3 Bacteriophage MS2 Preparation and Enumeration

MS2 stock was prepared as previously described.^29^ Briefly, phages were propagated using the host *Escherichia coli* followed by the serial purification steps of centrifugation and membrane filtration to remove bacterial cell debris, and ultracentrifugation to collect the phages. A double-agar layer method was used for the enumeration of infective MS2 particles.^30^ Electrocoagulated phage-containing suspension was filtered with a 0.45-μm polyethersulfone syringe filter before the enumeration, and the infective MS2 concentration in the filtrate was considered a bulk concentration.

### 2.4 Analytical Methods

Total iron concentration was measured following the HACH 8112 method where a purple-colored complex between Fe(III) and 2,4,6-tris(2-pyridyl)-s-triazine is quantified based on absorbance at 590 nm wavelength.

### 2.5 Calculations

Electrical energy (*E*, W h) consumed for electrolysis during EC was obtained by integrating the incremental energy consumption considering cell voltage changes. Energy consumption was calculated by multiplying current (*I* = 0.05 A), voltage at a given time (*V*(*t*), V), and time interval (Δ*t* = 1 s) and dividing by 3600 s/h (eq 1). Faradaic efficiency was estimated based on the ratio between the theoretical iron dosage calculated using Faraday’s law (eq 2) and the experimentally measured iron concentration. In eq 2, *m* (g) is the mass of iron, *A*_*W*_ is the atomic weight of iron (55.8 g/mol), *I* is the electrical current (A), *t* is the total electrolysis time (s), *z* is the number of electrons transferred (known to be 2),^31^ and *F* is the Faraday’s constant (96,485 C). MS2 log reduction value (LRV) was calculated by taking the common logarithm (base 10) of the ratio between the infective MS2 concentration added before EC (*N*_0_, PFU/mL) and that measured at a given time (*N*_*t*_, PFU/mL) as shown in eq 3.
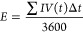
1
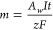
2
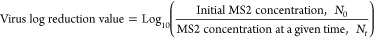
3

### 2.6 Optical and Electron Microscopy

The electrode surface was probed using an optical microscope (BX-53, Olympus). Additionally, scanning electron microscopy with energy-dispersive X-ray spectroscopy (SEM-EDS) was employed to examine surface morphology and elemental composition (Merlin, Carl Zeiss AG).

### 2.7 Neutron Tomography

Neutron tomography of porous electrodes was conducted at the High Flux Isotope Reactor (HFIR) of the Oak Ridge National Laboratory.^32,33^ The Multimodal Advanced Radiography Station (MARS) instrument was set to measure at L/D = 600, where L is the distance from the aperture to the detector (6.59 m) and D is the aperture diameter (11 mm). It is noted that the overall dimensions of the porous electrodes used for neutron tomography were different from the ones for the batch experiment described in Section 2.1 because the field-of-view available for the scanning was limited to an area of approximately 8.6 cm × 8.6 cm. Hence, 10, 50, and 100 PPI porous cylindrical electrodes of 4 cm diameter and 1 cm thickness were employed. Four electrodes were alternately stacked for scanning with aluminum plates as spacers to give a clear contrast between the porous iron electrodes needed for the subsequent image processing. The neutrons in the aperture defined beam traveled through helium filled flight tubes with beam scrappers and passed through the object to be scanned. Neutrons that were not absorbed nor scattered were then captured by a neutron scintillator screen, ^6^LiF/ZnS with 100-μm thickness, to convert neutron signal into visible light. Then a lens coupled charge-coupled device (CCD) was used to collect a 2D image, resulting an effective pixel size of 42 μm. During scanning, the stack of samples was incrementally rotated from 0° to 360° by 0.42°. 3D reconstruction of collected scans was performed using in-house developed iMars3D and rockit that utilizes TomoPy,^101^ Algotom,^102^ and bm3d-streak-removal.^103^ Fast Filtered Back Projection (FFBT) or Gridrec^104,105^ was used in this case.^34^ Visualization and data analysis were performed using Amira.^35^

**Figure 1:**
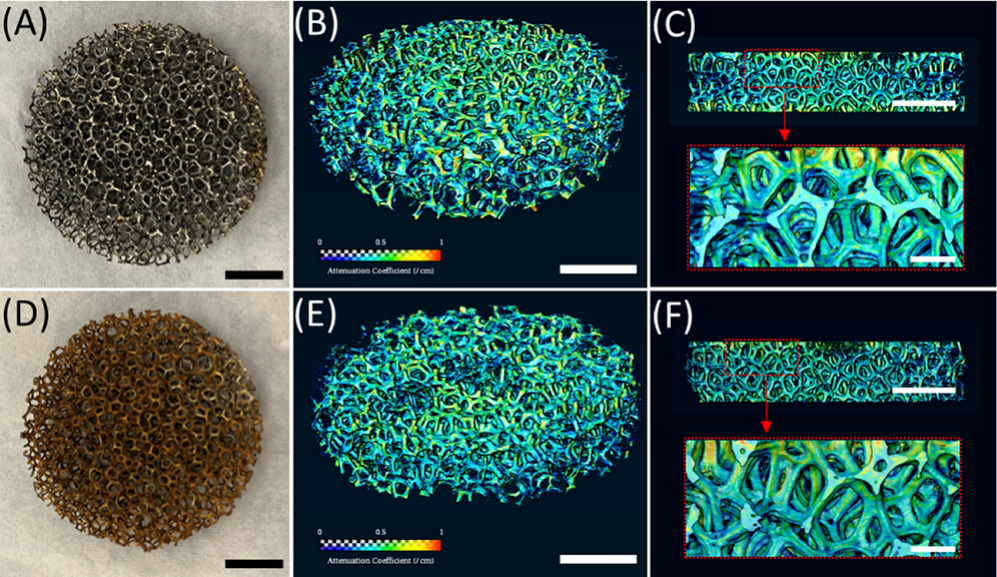
Representative neutron tomograms of porous cylindrical electrodes. A pristine 10 PPI electrode (A, scale bar: 10 mm) was neutron-scanned and three-dimensionally reconstructed (B, scale bar: 10 mm). The neutron attenuation coefficient was represented in a color spectrum scaling from 0 to 1. The reconstructed 3D image enabled internal pore navigation (top image of C, cross-section at the concentric center of B, scale bar: 10 mm) and a local visualization (bottom image of C, zoomed-in image of the red-dotted box of the top image, scale bar: 2 mm). (D, E, and F) Corresponding images of the same electrodes after electrolysis at 1.0 A for 40 min.

A liter of synthetic secondary effluent at pH 6.5 ± 0.2 was electrocoagulated in a batch mode using a pair of porous cylindrical electrodes placed 1 cm apart from each other (Figure S1). Electrolysis was conducted at a higher current of 1.0 ± 0.1 A and a longer time of 40 min compared to batch experiments described in earlier sections to accelerate electrode surface alterations. Afterward, the electrodes were gently blotted to remove excess solution and oven-dried at 60 °C in a vacuum for 4 h as a preparation step for neutron scanning. Figure 1 shows photos of pristine and used cylindrical 10 PPI electrodes (Figures 1A and 1D, respectively) with corresponding volumetric tomograms (Figures 1B and 1E). The colored surface illustrates a neutron attenuation coefficient ranging from 0 (purple) to 1 (red). The resulting tomograms provided information on the spatial distribution of volume and neutron attenuation coefficient not only of the outer layer but also of the inner structures (Figures 1C and 1F).

**Figure 2:**
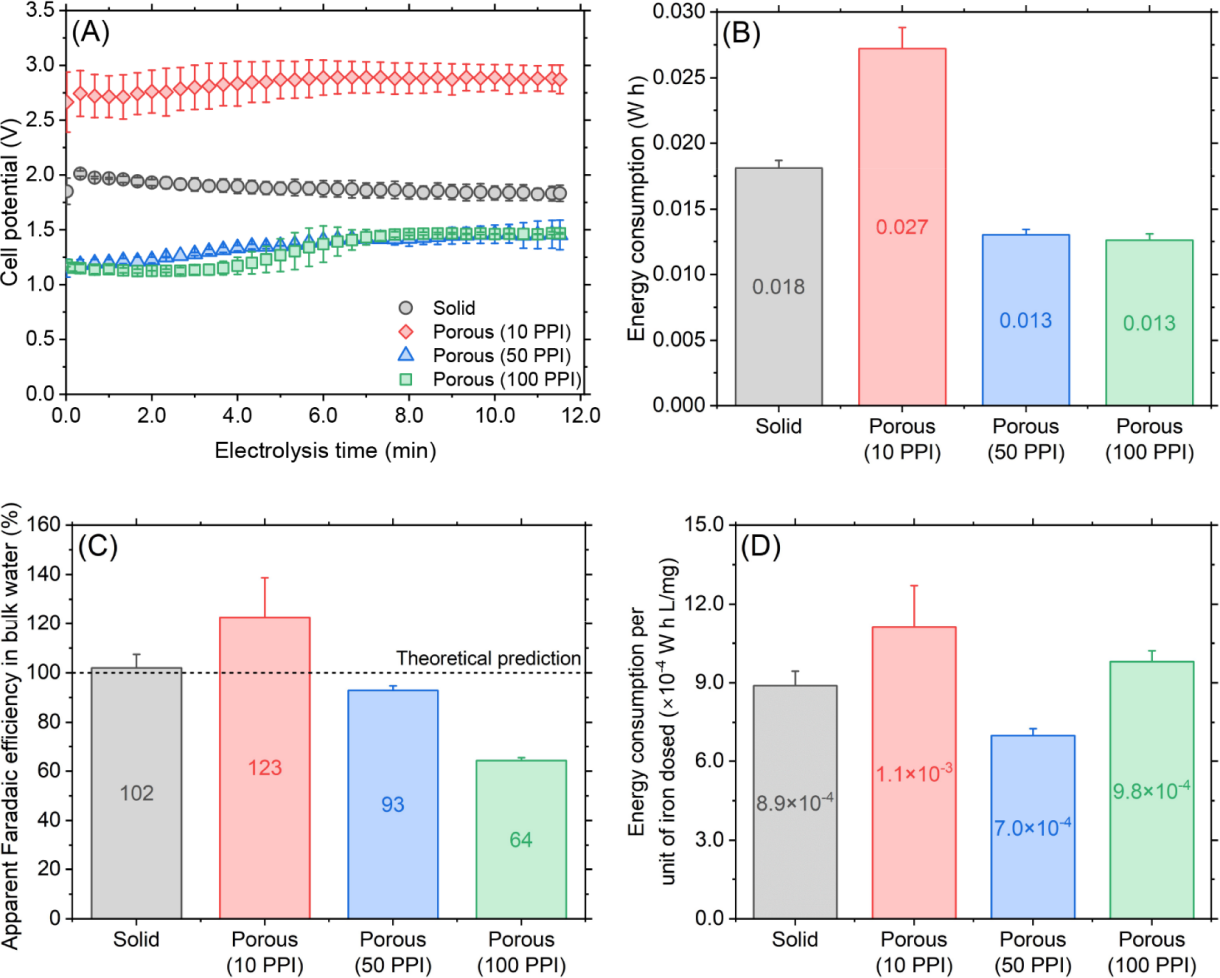
Electrochemical behavior comparison of solid and porous iron electrodes when electrolysis was performed at 0.05 A for 691 s. (A) Cell voltage history during electrolysis (data points every 20 s are shown so as to clearly see individual data points, error bars, and the overall trend). (B) Electrical energy consumption for electrolysis (all data points measured every second were used for the calculation following eq 1). (C) Faradaic efficiency calculated based on the measured total iron concentration in bulk water and theoretical prediction using Faraday’s law. (D) Electrical energy consumed for unit iron dosing.

## 3 Results and Discussion

### 3.1 Electrochemical Behavior of Solid and Porous Iron Electrodes

A half-liter of synthetic secondary effluent at pH 6.5 was electrocoagulated using porous anodes and cathodes to compare with their solid counterparts. A dosage of 20 mg/L total iron in the bulk water was targeted by electrolyzing the anode at a constant current of 0.05 A for 11.5 min. Figure 2A compares temporal cell potential values during the electrolysis using different types of electrodes. In the solid electrode configuration, the cell voltage abruptly increased from 1.85 to 2.02 V during the initial 20 s followed by a gradual decrease reaching 1.83 V at the end of the electrolysis. The initial cell potential surge was indicative of an immediate formation of passivating oxide layers upon the electrolysis,^36^ which was soon broken down by “pitting promoters” such as chloride.^37^ Further, the incremental potential decline thereafter was possibly due to the increase of surface area by pitting corrosion on the anode. Overall, the electrical energy consumed during the electrolysis was calculated as 0.018 W·h. Interestingly, the least porous electrode (i.e., 10 PPI) resulted in a higher cell potential (Figure 2A) and energy consumption (0.027 W·h, Figure 2B) compared with the solid electrode configuration, whereas the other two porous electrodes (i.e., 50 and 100 PPI) required lower cell potentials (Figure 2A) consequently consuming lower energies (Figure 2B). The higher cell potential with the 10 PPI electrodes was possibly due to the lower surface area compared to that of higher-porosity electrodes combined with an increased electrical resistance because of the presence of H_2_ gas bubbles attached on and/or trapped in cathode pores.^38−40^ It should be also noted that regardless of the porosity, the cell potential of all porous electrodes appeared to increase at the end of electrolysis indicating electrode fouling,^41^ which will be explored in the following sections.

Figure 2C compares Faradaic efficiency for different electrodes based on iron concentrations measured in the bulk solution. A nearly 100% efficiency of solid electrodes demonstrated that virtually all electrodissolved iron was delivered to the bulk solution as intended. With the 10 PPI porous electrodes, a Faradaic efficiency greater than 100% with a relatively large standard deviation (coefficient of variation = 0.13) was obtained, which was seemingly due to degrading structural integrity during the electrolysis. As shown Figure S2, the 10 PPI porous anode after electrolysis featured several abnormally large voids (white-dotted circles) suggesting a possible nonelectrolytic detachment of local branches that were sparsely connected to the main network, thus, a concern of structural instability. Meanwhile, 50 PPI porous electrodes achieved a 93% Faradaic efficiency indicating a slight absence of electrodissolved iron from the bulk water, which was attributed to iron floc deposition onto the anode and cathode surface internal to the electrodes (middle column Figure S2). The most porous electrode (100 PPI) resulted in only 64% Faradaic efficiency, thus a severe waste of electrical energy. This behavior was rationalized by a significant amount of iron flocs found especially on the cathode, clogging the pores (red-dotted circles Figure S2). In summary, the porous electrodes with 50 PPI were found to be optimal in terms of energy efficiency to deliver electrolyzed iron into the bulk water (Figure 2D).

### 3.2 Improved Energy Efficiency of Virus Attenuation with Porous Electrodes

**Figure 3:**
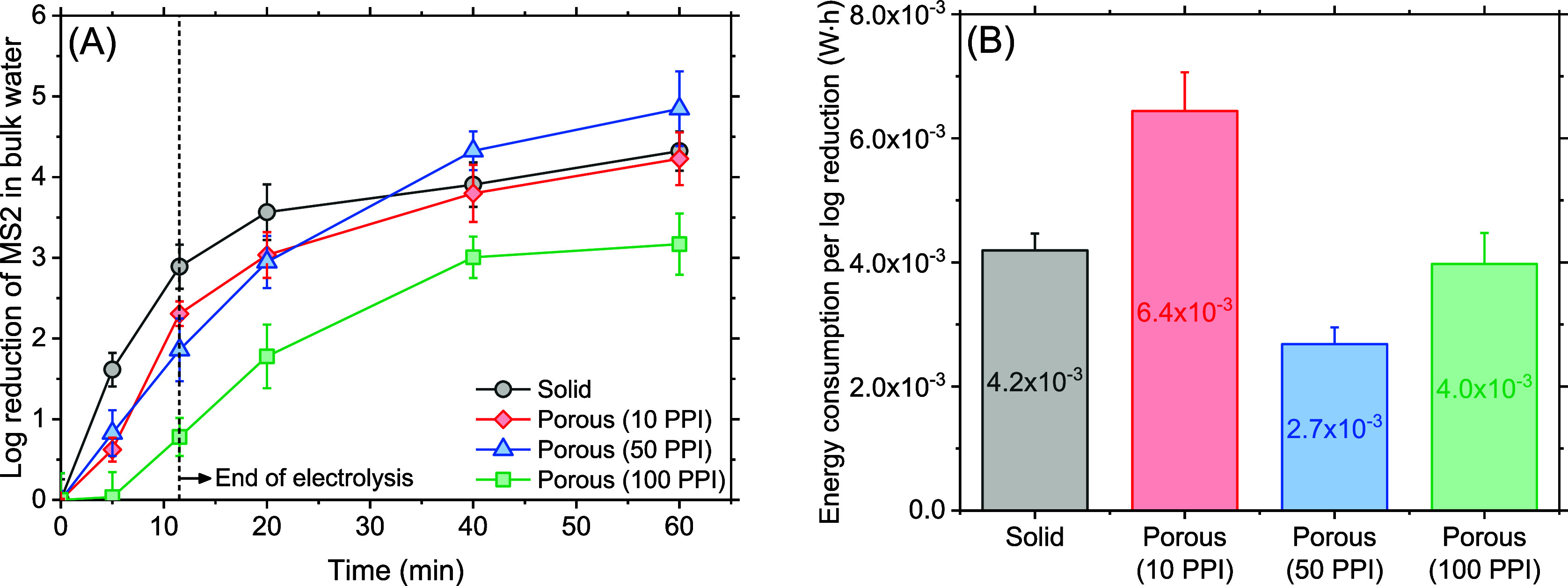
Improved virus control performance with 50 PPI porous electrodes. (A) Log reduction of MS2 in bulk water over time. (B) Electrical energy required for a unit log reduction of MS2 at *t* = 60 min.

The performance of different iron electrodes was also evaluated in terms of virus log reduction values. Synthetic secondary effluent at pH 6.5 containing MS2 phages at ∼10^7^ PFU/mL was electrocoagulated at 0.05 A for 11.5 min with rapid mixing aiming to electrodissolve 20 mg/L iron in total. After electrolysis, the resulting suspension was slowly mixed (i.e., flocculated) for the next 48.5 min. Figure 3A depicts temporal profiles of MS2 log reduction in bulk water wherein the solid and two porous electrodes (10 and 50 PPI) showed similar trajectories achieving 4.3 ± 0.3, 4.2 ± 0.2, and 4.8 ± 0.5 log reduction at the end of the experiment, respectively. In contrast, only a 3.2 ± 0.4 log reduction was obtained with the 100 PPI electrode. Given the virus attenuation mechanisms during iron EC essentially triggered by Fe(II) and Fe(III) species,^42^ this result was attributed to the lowest iron delivery efficiency of the 100 PPI electrodes found in the previous section. Also, it was unclear as to why the 10 PPI electrodes did not perform better despite their higher Faradaic efficiency (i.e., higher total Fe measured in bulk). As discussed earlier (Figures 2C and S2), a portion of iron detected in bulk might be introduced as elemental iron (i.e., Fe^0^) via a non-Faradaic mechanism which, if so, was not likely to act as a coagulant. The energy efficiency for a unit log reduction of MS2 shown in Figure 3B again confirmed that the electrodes with an intermediate porosity (50 PPI) outperformed the others emphasizing the porosity as a critical factor in energy efficient EC operation.

It is emphasized that the principal focus of this manuscript is to compare the relative performance of porous electrodes with respect to their solid counterparts. Nevertheless, it is worth mentioning that we have recently demonstrated that MS2 attenuation during iron EC is the combined result of sweep coagulation and inactivation^29,43^ (mainly driven via indirect oxidation by reactive oxygen and higher valent iron species in the bulk phase produced via electro-Fenton reactions.^8,10,29,43^

### 3.3 Optical Microscopic Investigation of Electrode Surface

**Figure 4:**
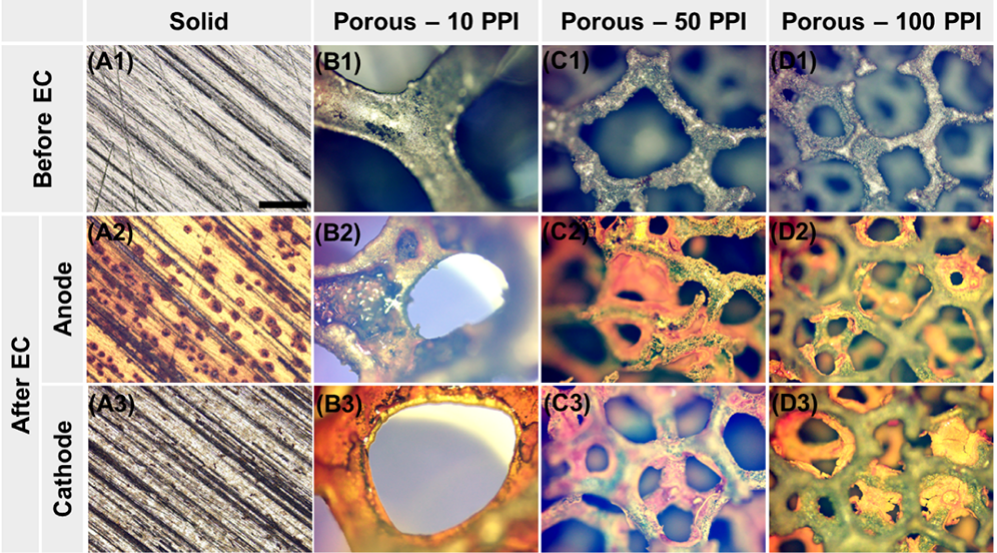
Optical microscopy of surface morphology changes on solid (A1–A3) and porous Fe electrodes (B1–D3) after electrolysis at 0.05 A for 11.5 min at pH 6.5. The scale bar in A1 represents the 500-μm length and applies to all the images. Note that Figures 4D2 and 4D3 were intentionally focused on the clogged inner pore layer, resulting in blurred images of clean outer pore layers.

Optical microscopy revealed visually distinguishable changes in the outer (pore) structure of the electrodes. The solid electrodes before EC displayed a planar silver surface with unidirectional scratches probably originating from the manufacturing process (Figure 4A1). After electrolysis for 11.5 min (i.e., used as an anode), a number of circular pits of approximately 10–50 μm diameter appeared on the electrode surface^44^ (Figures 4A2 and S3A) indicating pitting corrosion, i.e., localized breakdown of a passivating metal oxyhydroxide layer assisted by anions such as chloride, thereby facilitating continuous electrodissolution.^45^ As the pits developed inward during electrolysis,^46,47^ the surface area available for iron dissolution increased, resulting in a gradual cell potential decline as observed in Figure 2A for the solid electrodes. The color change of the nonpitted area into yellowish brown was indicative of Fe(III) oxyhydroxide such as ferrihydrite and lepidocrocite,^24^ which could cause a slight loss of iron delivery to bulk water. In contrast to the anode, the cathode only showed marginal morphological changes with patchy light-brown precipitates (Figures 4A3 and S3B). These precipitates could be Fe(III) oxyhydroxide that was deposited on the cathode from the bulk water and/or was formed via chemical dissolution of iron from the cathode due to a locally high pH in the vicinity of the cathodic surface, as it is visually evidenced in Figure S4A.^48,49^ Overall, the visual observation of the solid electrodes before and after EC at the macroscale indirectly supported the cell potential decline and the near 100% Faradaic efficiency discussed earlier.

The 10 PPI porous electrodes demonstrated a similar behavior to the solid ones when used as anode. The smooth surface of the pristine iron struts (Figure 4B1) became rough with irregular voids, clearly indicating localized electrolytic iron dissolution (Figure 4B2). In contrast to the solid plate cathode, the 10 PPI porous cathode was severely covered with a layer of orange-colored precipitates (again, presumably ferric oxyhydroxide).^41^ Though it is not yet fully clear, the chemical dissolution mentioned earlier appeared to be accelerated as pit-like features were occasionally found on the cathode surface (Figure S4B). Hence, the 10 PPI electrode configuration seemed to concurrently experience the “cell-potential-reducing” pitting corrosion on the anode and the “cell-potential-elevating” cathode passivation as well as the possible electrical insulation effect of H_2_ bubbles.^38−40^ The former dominated the initial phase of electrolysis while the latter overwhelmed in the terminal phase as indicated by the cell potential profile with an inflection point (Figure 2A). Finally, pore blockage was not observed in the 10 PPI electrodes suggesting that the transport of iron coagulants was not likely hindered while passing through the pores. The 50 PPI anode, with a moderate surface deposition (Figure 4C2), did not show circular pits or voids on its surface. It was speculated that struts became irregularly thinner and even disconnected as the local pits grew (Figure S3C). Also, similar to the 10 PPI cathode, pore blockage was not evident in the 50 PPI cathode (Figures 4C3 and S3D; see also Figure S4C). Therefore, the cell potential increase (Figure 2A) was attributed to the surface coverage by orange-colored Fe(III) precipitates of both anode and cathode. In contrast, the most porous electrode (100 PPI) showed clear signs of pore blockage, as well as considerable surface coverage (Figure S4D). Figure 4D2 depicts an inner pore of 100 PPI anode clogged with a layer of orange precipitates while Figure 4D3 emphasizes harshly blocked pores found in the cathode. The important role of hydrodynamic mixing within the pore structures was hinted at by the very outer layers of both 100 PPI electrodes largely remaining intact (Figures S3E and F). The cell potential declined due to the higher surface area compared to that of solid electrodes (Figure 2A), and the poor Faradaic efficiency of the 100 PPI electrode (Figure 2C) was attributed to the surface coverage of both electrodes and the hindered iron transport from the anode to the bulk solution.

Since EC generates soluble Fe(II),^43^ cathodic H_2_O_2_ production and associated electro-Fenton reactions^43^ as well as dissolved oxygen^50^ would have oxidized it^10^ to insoluble Fe(III) in the bulk water to cause pore blocking in the case of the porous electrodes. Free chlorine was always below detection limits (<0.2 mg/L, HACH Method 8021).

### 3.4 Probing Internal Structures using nCT

**Figure 5:**
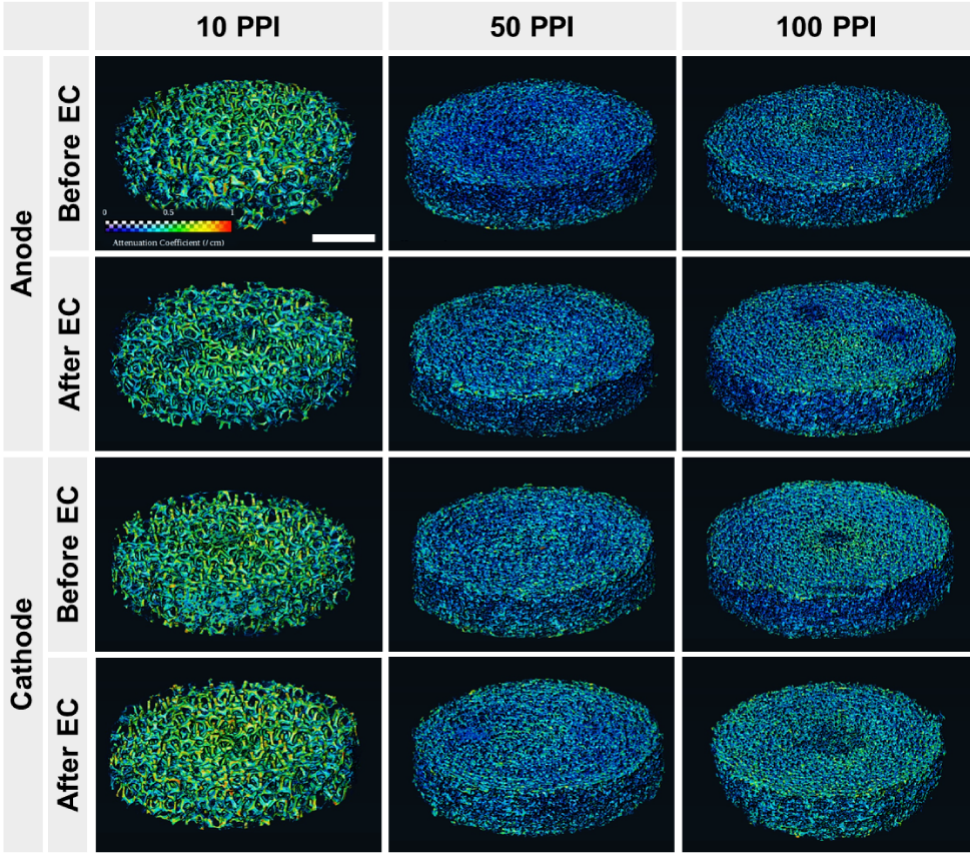
Neutron tomography of cylindrical porous electrodes. EC was performed at 1.0 A for 40 min. Scales of attenuation coefficient (0–1/cm) and physical length (10 mm) shown in the top left panel apply for all the images.

Changes in the internal structure of porous electrodes induced during EC were investigated using nCT as summarized in Figure 5. The porous electrodes before EC appeared in (bright) blue overall indicating relatively low neutron attenuation by iron (0.1–0.2/cm). Green- and yellow-colored areas (attenuation coefficient approximately 0.5–0.7/cm) of both 10 PPI anode and cathode even before the EC suggested atmospheric corrosion forming iron oxyhydroxides containing H,^51^ a highly neutron attenuating element.^52^ After electrolysis for 40 min, the 10 PPI anode appeared with noticeable pits near the center while the yellow-, green- and red-colored areas (i.e., strong neutron attenuating regions) increased in the cathode. This result was consistent with the optical microscopy observations wherein the volume loss of the anode and surface deposition of ferric oxyhydroxides on the cathode were found (Figure 4). For both 50 and 100 PPI, EC increased brighter areas in the anode and cathode, again suggesting the deposition of ferric oxyhydroxide layers. However, regardless of the substantial differences in iron delivery performance by the porous electrodes, visual differences in the exterior of nCT-reconstructed images were low and relatively indiscernible. Further, the notable changes in volume and attenuation coefficient of the entire porous electrodes (Section S7) strongly suggested the need for internal structure investigation.

**Figure 6:**
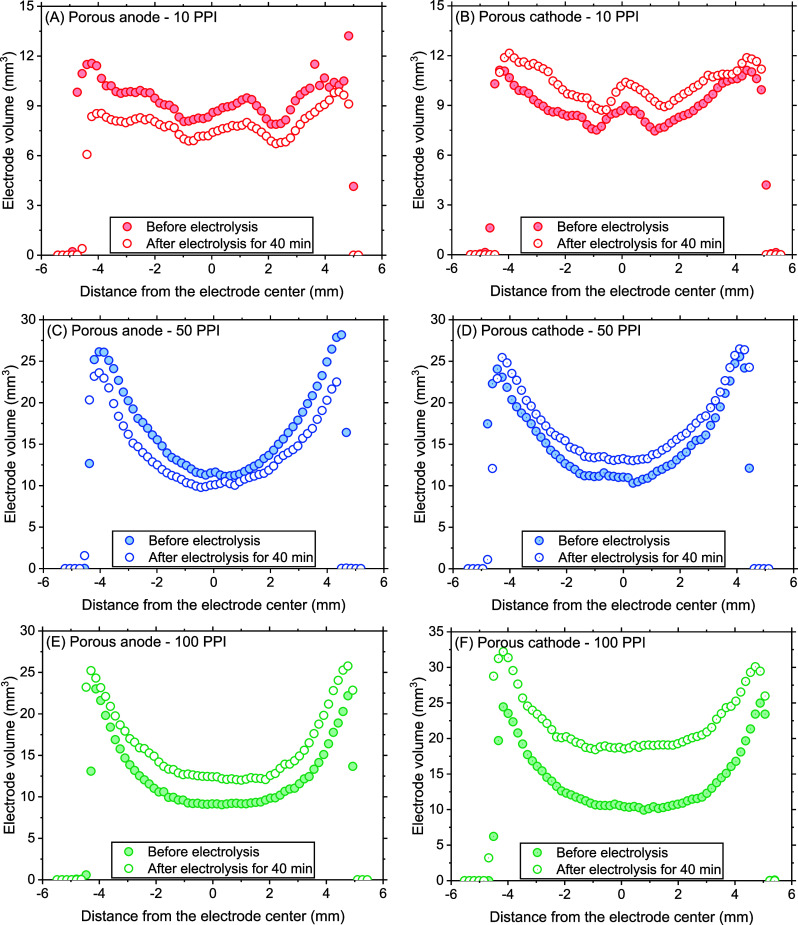
Porous anode and cathode volume across the thickness estimated based on neutron tomograms before and after 40 min electrolysis.

In order to resolve the spatial distribution of quantified changes in the volume and neutron attenuation coefficient, these parameters were plotted across the electrode geometry. Figure 6 depicts the electrode volume change across the electrode thickness. The 10 PPI anode volume decreased overall after electrolysis, whereas the opposite was observed with the cathode as shown in Figures 6A and 6B, respectively, in agreement with the results discussed in earlier sections. However, the profile based on nCT also provided another important piece of information; relatively uniform volume changes along the thickness (see also Figure S8A) suggest uniform usage of the anode and evenly distributed deposits on the cathode.

A slightly different behavior was found in the 50 PPI anode; its volume decreased to a larger extent at both ends of the electrode (Figures 6C and S8C). It was hypothesized that either the hindered mass transport near the electrode center facilitated iron precipitate accumulation compensating the volume loss due to the electrolysis, while iron dissolved from the outer electrode layers successfully escaped to the bulk, or simply the electrolysis was only promoted near the geometric boundary with the electrolyte. Similarly, the overall volume increase of the 50 PPI cathode by 12% presented in Figure S5A was found nonuniformly distributed, with slightly more accumulation in the center (Figures 6D and S8C), indicative of hindered mixing in the internal pores. This could not only cause the deposition of iron precipitates but also a poor pH-mitigating effect that accelerates a chemical Fe dissolution near the highly basic cathodic surface. A more drastically skewed volume change in 100 PPI anode and cathode further pronounced the crucial impact of porosity. Substantial volume increase was concentrated near the center and middle of both 100 PPI electrodes (Figures 6E, 6F, and S8E) implied the facilitated accumulation of iron precipitate due to poor mixing within the internal pores.

**Figure 7:**
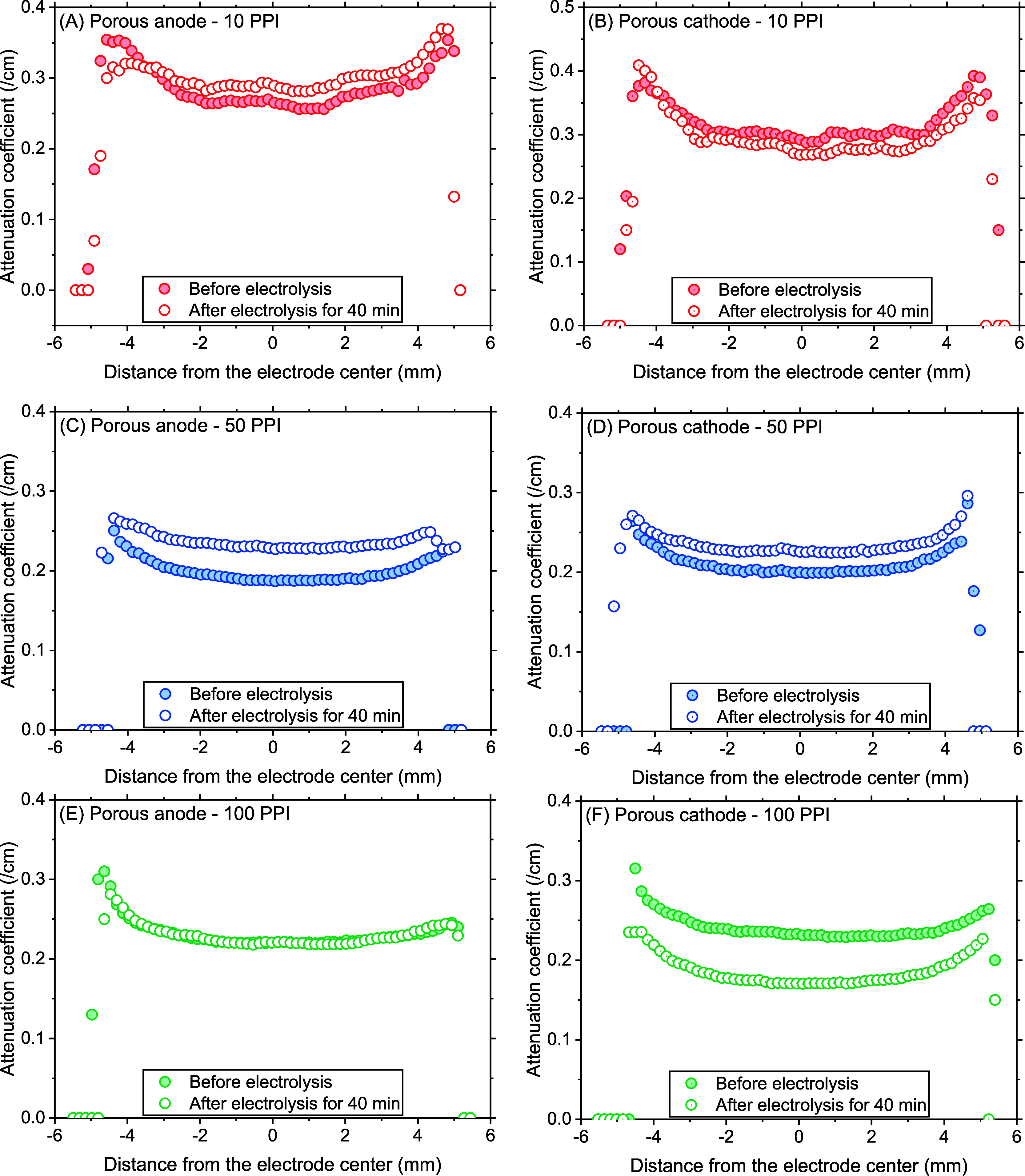
Porous anode and cathode attenuation coefficient across the thickness estimated based on neutron tomograms before and after 40 min electrolysis.

**Figure 8:**
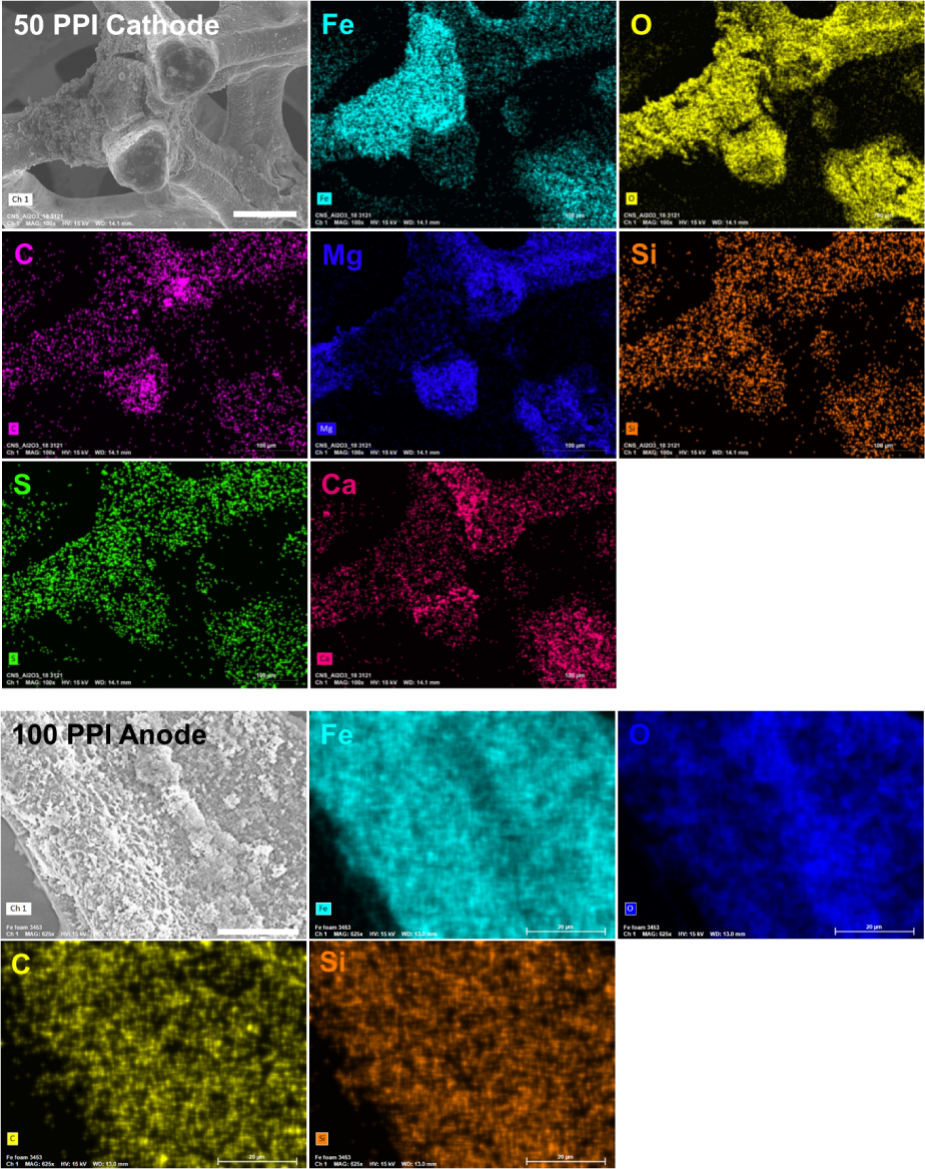
SEM images and corresponding EDS results of selected cylindrical electrodes after 40 min electrolysis. Top panels: Cylindrical porous 50 PPI cathode (scale represents 100 μm and applies to all the EDS images). Bottom panels: Cylindrical porous 100 PPI anode (scale represents 20 μm and applies to all the EDS images).

The spatial distribution of the attenuation coefficient found for the 10 PPI electrodes (Figures 7A and 7B) followed the same trend as the volume, i.e., insignificant differences along the electrode thickness (see also Figure S8B). Interestingly, unlike the volume change profile, the attenuation coefficient change for the 50 PPI anode was slightly center-concentrated, strongly suggesting the iron precipitate accumulation in the internal pores (Figures 7C and S8D). Therefore, a lower volume decrease in the center of the 50 PPI anode was more likely due to the iron precipitate accumulation compensating for the volume loss due to the electrolysis, rather than a locally promoted electrolysis at both ends of the electrode near the electrolyte. Similarly, the center-concentrated volume increase of the 50 PPI cathode was attributed to iron precipitate deposition (Figure 7D). Also, the lesser extent of cathode attenuation compared to that of the anode again inferred substantial deposition of low-attenuating elements such as Ca, Mg, C, O, and Si (top panels in Figure 8). The marginal overall changes of the attenuation coefficient in the 100 PPI anode (Figure S5B) were spatially uniform (Figures 7E and S8F) unlike the volume change distribution. We attributed this difference to severe iron corrosion accompanied by the deposition of various elements. Meanwhile, a substantially declined overall coefficient of 100 PPI cathode (Figure S5B), possibly due to the low neutron-attenuating elements such as C, O, and Si as hinted by the case of 100 PPI anode (bottom panels in Figure 8), showed a trend with notable changes along the thickness with a slight emphasis on the center (Figure 7F), similar to its volume profile. Given the severely hindered mixing conditions across the internal pore structure, it was not likely that these elements were delivered from the bulk phase into the internal pores. Rather, it is more convincing that these elements, initially existing in the cathode pores before electrolysis, were captured by precipitates generated following chemical dissolution of iron.

### 3.5 Additional Energy Savings in Flow-Through Operation

**Figure 9:**
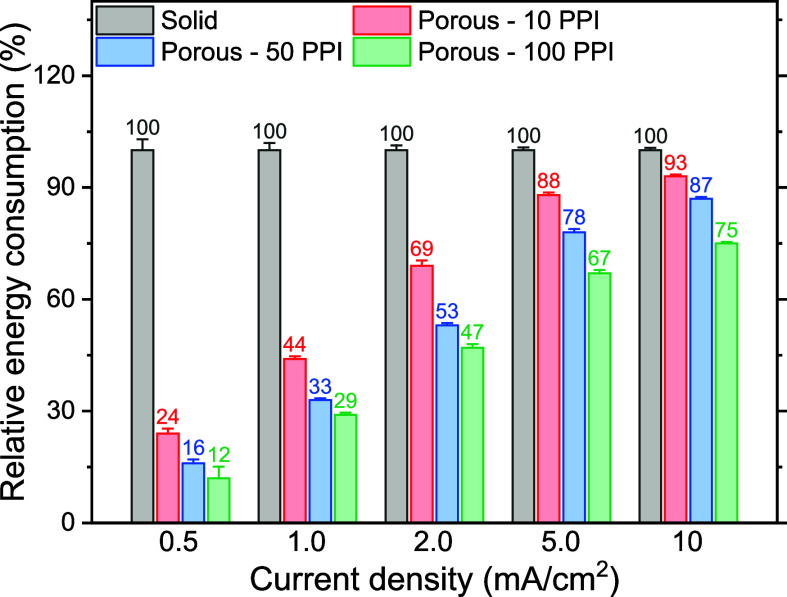
Relative electrical energy consumption with porous electrodes at different current densities with respect to the solid electrode when EC was performed in a flow-through operation. Averages and standard deviations are represented.

Electrical energy consumption by solid and porous electrodes was further compared when EC was performed in a flow-through reactor (Figure S9). Similar to batch operation, the electrodes were placed 1 cm apart. Synthetic secondary effluent at pH 7.7 ± 0.4 was passed through the reactor at 500 mL/min, while electrolysis was conducted at a constant current density ranging from 0.5 to 10 mA/cm^2^. The average cell potential monitored during the electrolysis for 5 min was used to calculate the electrical energy consumption (note that the total iron dose was not controlled). Figure 9 summarizes the relative energy consumption using porous electrodes with different pore sizes with respect to the solid electrode.

As shown, all porous electrodes demonstrated energy efficiency improvement, notably with 100 PPI requiring only 12% of the energy used by the solid electrodes (i.e., 88% reduction) at the lowest current density of 0.5 mA/cm^2^. Interestingly, batch operation results at 0.95 mA/cm^2^ shown in Figure 2B showed that 10 PPI electrodes required more energy than solid electrodes and 50 and 100 PPI electrodes that consumed similar amounts of energy. In contrast, the 10 PPI electrode was found to be more energy-efficient than solid electrodes and 100 PPI was found to be most energy-efficient when used in a flow-through operation at a similar current density of 1.0 mA/cm^2^. Meanwhile, 50 and 100 PPI electrodes saved more energy compared to the batch mode (energy savings: 28% → 67%, and 28% → 71%, respectively). Furthermore, batch EC was performed using the flow-through reactor system to better clarify the importance of mixing. As shown in Figure S10, at all current densities explored, energy consumption increased, some of which even required higher electrical energy than that needed for the solid electrodes. These results demonstrate the crucial role of fluid mixing within pores to alleviate the electrode passivation observed in the earlier sections. The deteriorating performance of porous electrodes with increasing current density was attributed to accelerated surface deposition of solids at higher current densities. However, since the total iron dosage was not controlled, lesser amounts of iron were introduced at lower current densities. The effects of lower iron dosages on electrode fouling should not be neglected. Therefore, further investigation considering the total amount of iron dosed is necessary to better understand how electrode porosity and current density affect flow-through EC system performance and clarify these parameters for design alongside coagulant dose and retention time.

## 4 Summary and Conclusions

Major observations for EC using porous iron electrodes obtained from this investigation are summarized in Table 2. Results to an optimal porosity for energy savings. In batch experiments, 50-PPI porous electrodes of intermediate porosity were the most energy efficient compared to solid (nonporous), 10-PPI (low porosity), and 100-PPI (high porosity) electrodes. The same trend was observed for virus log reduction values, where the beneficial application of iron electrodes with an optimal porosity (50 PPI in this case) was demonstrated. Optical microscopy provided evidence of severe pore-clogging by Fe precipitates exclusively on the inner surface of the highest porosity electrodes. Investigation of the internal pore structure using a nondestructive nCT revealed that mass transfer of iron dissolved at the anode could be hindered, causing poor apparent Faradaic efficiency (measured using bulk iron concentrations). At the same time, severe accumulation of iron precipitates on the internal surface of the cathode caused clogging of the pores, detrimentally affecting the process energy efficiency. Finally, flow-through EC provided promising results using porous electrodes with energy savings of up to 88% compared to solid electrodes.

**Table 2:** Summary of Electrode Alteration and Operational Performance

	Electrode porosity			
Parameter	Nonporous (solid)	Low (10 PPI)	Intermediate (50 PPI)	High (100 PPI)
Anode alteration	Evident pitting corrosion	Evident pitting corrosion	Evident pitting corrosion	Severe internal pore clogging
	Slight surface deposition	No pore clogging	Moderate surface deposition	Moderate surface deposition
		Structural instability		
		Uniform usage of outer and inner pores.	Insignificant pore clogging	Moderate pore clogging
Cathode alteration	Slight surface deposition	Severe surface deposition	Moderate surface coverage	Severe surface coverage
		No pore clogging	Insignificant internal pore clogging	Severe internal pore clogging
Faradaic efficiency of iron dosing	As predicted (∼100%)	Super-Faradaic (>100%)	As predicted (∼100%)	Sub-Faradaic (<100%)
Energy consumption	Moderate	High	Low	Low
Overall performance	Moderate	Poor	Best	Moderate

Overall, this study demonstrated energy savings using porous iron electrodes compared to solid electrodes. For batch EC, there was an optimal porosity needed to overcome mass transfer limitations. For continuous-flow EC, which can be potentially scaled up for industrial applications, a flow-through-electrodes configuration eliminated mass transfer limitations both at the anode and cathode, demonstrating enhanced benefits of porous electrodes with respect to energy savings. Porous electrodes employed for EC in this study also showed advantages over solid electrodes for virus attenuation. The energy and cost of manufacturing porous electrodes, however, must be carefully evaluated by technoeconomic and life cycle assessments before they can be considered for industrial (waste)water treatment. Due to the nature of the process that utilizes sacrificial electrodes, replacement cost may account for 16–78% of the total operation expenses (OpEx).^53 −57^ Given the high cost of porous electrodes, ranging from $7 to $12,000 per kg depending on the material and manufacturing process,^58 −60^ compared to planar counterparts ($0.3–$5.6 per kg),^53 −55,57,61^ the initial and replacement costs of porous electrodes could be substantially higher in the long term. Therefore, further reduction in the price of porous electrodes is necessary, which can be achieved by mass production, manufacturing process optimization, utilizing scrub metals, etc.^62 −64^ Also, since this work was conducted with synthetic water without any organic substances following the general approach taken to understand the fundamental capabilities of EC,^65 −67^ this study needs to be extended with real-world waters containing organic matter to draw more meaningful conclusions for a scale-up.

## Supporting Information Available

Synthetic secondary effluent solution composition, details of the electrolysis time calculation, a photo of a batch electrocoagulation setup with cylindrical porous electrodes, photos of porous foam electrodes after electrocoagulation, optical microscopic images of solid and porous foam electrodes after electrocoagulation, quantitative analysis using nCT of cylindrical porous electrodes and additional discussion, mass change of the cylindrical porous electrodes, SEM images of the cylindrical porous electrode (100 PPI), relative changes of electrode volume and attenuation coefficient estimated by nCT, a photo of a flow-through electrocoagulation system and a control experiment for energy consumption comparison (PDF)
