# Supplementary material for: Porous Iron Electrodes Reduce Energy Consumption During Electrocoagulation of a Virus Surrogate: Insights into Performance Enhancements Using Three-Dimensional Neutron Computed Tomography
Source: ACS ES T Eng. 2024 Sep 23;4(10):2573–84. doi: 10.1021/acsestengg.4c00317 (PMC11474953; doi:10.1021/acsestengg.4c00317)
Supplement: Supplementary file 1 — ee4c00317_si_001.pdf [file ee4c00317_si_001.pdf]

Supporting Information

Porous Iron Electrodes Reduce Energy Consumption During Electrocoagulation of a Virus Surrogate: Insights into Performance Enhancements Using Three-Dimensional Neutron Computed Tomography

Kyungho Kim[1,*], Cesar Castillo[2], Gyoung G. Jang[2], Yuxuan Zhang[3], Costas Tsouris[2], Shankararaman Chellam[1]

[1]Department of Civil & Environmental Engineering, Texas A&M University, College Station, Texas 77843, United States

[2]Manufacturing Science Division, Oak Ridge National Laboratory (ORNL), Oak Ridge, Tennessee 37831, United States

[3]Neutron Scattering Division, ORNL, Oak Ridge, Tennessee 37831, United States

[*]Corresponding author: Kyungho Kim, Email: kyunghokim@tamu.edu

Section S1. Synthetic Secondary Effluent Composition. The synthetic solution used in this study was prepared to closely simulate the actual secondary effluent water of two case studies as shown below (Table S1).

Table S1. Water composition of two case studies of secondary effluent reuse and the formulated model water for this project.

Parameter	Case 1[1]	Case 2[2,3]	Synthetic secondary effluent in this study
Chloride (mg/L)	289	285±21	346
Sulfate (mg/L)	268	195±20	200
Calcium (mg/L)	78.3	79.7±4.5	75
Magnesium (mg/L)	18.8	28.6±2.9	25
Silica (mg/L)	45.0	20.3±1.5	30
Sodium (mg/L)	368	225±15	225
Hardness (mg/L as $CaCO_3$)	253	317±21	291.7
Alkalinity (mg/L as $CaCO_3$)	110	202±15	59.3
pH	7.0	7.2±0.1	6.5
Conductivity (μS/cm)	-	1680±80	1550

Section S2. Electrolysis Time Calculation. The electrolysis time of 11.5 min to aim 20 mg/L Fe in 0.5 L synthetic water at 0.05 A was obtained based on Faraday's Law for batch EC Experiments with solid and porous foam electrodes as shown below.

$$m = \frac{A_W \cdot I \cdot t}{z \cdot F}$$

where m is the mass of total Fe (g), A_W is the atomic weight of Fe (55.9 g/mol), I is the current (A or C/s), t is the electrolysis time (s), z is the number of electrons transferred (assumed to be 2)[4], and F is the Faraday's constant (96,485 C/eq). Rearranging and solving the equation for t for 0.01 g Fe (i.e., 20 mg/L for 0.5 L) with given parameter values yields,

$$t = m \cdot \frac{z \cdot F}{A_W \cdot I} = (0.01\ g) \cdot \frac{2 \cdot \left(96485 \frac{C}{mol}\right)}{\left(55.9 \frac{\mathrm{g}}{\mathrm{mol}}\right) \cdot \left(0.05 \frac{\mathrm{C}}{\mathrm{s}}\right)} = 690\ s\ or\ 11.5\ min$$

Section S3. Batch Electrocoagulation Set-up with Cylindrical Porous Electrodes for Neutron Tomography

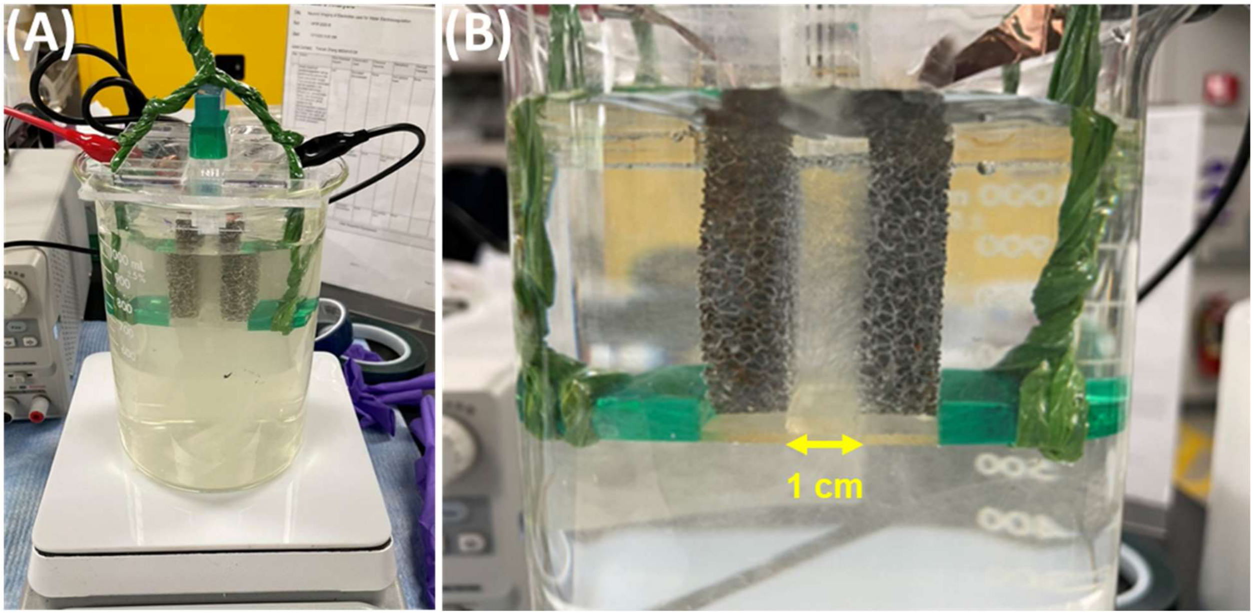

Figure S1. Photos of electrocoagulation with a set of porous cylindrical electrodes. The overall setup is shown in (A), and the vertical placement of the electrodes is emphasized in (B).

Section S4. Images of Porous Foam Electrodes after Electrolysis

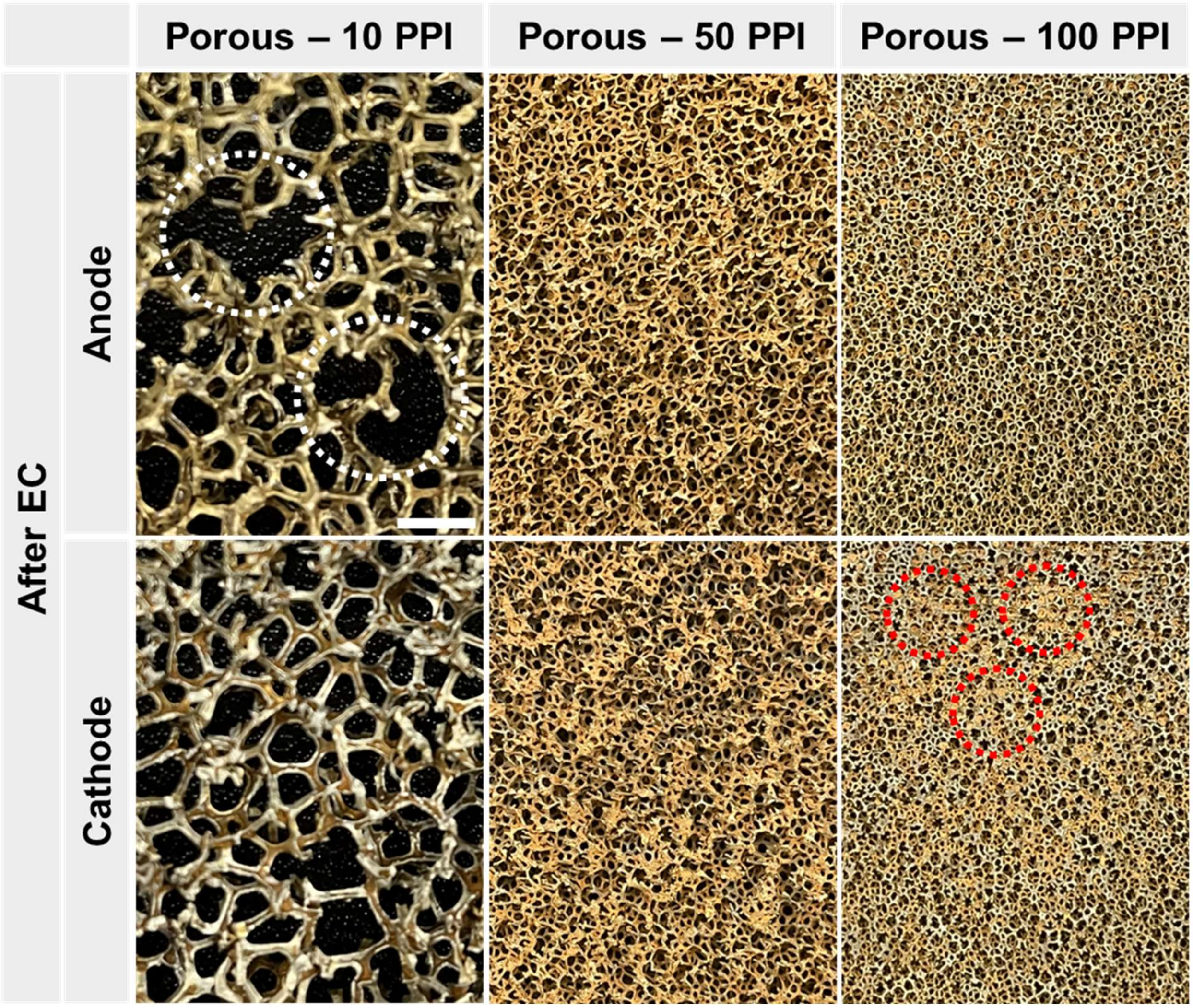

Figure S2. Structural changes of porous electrodes. The scale bar in the top left image represents 5 mm and is shared by the other images.

Section S5. Additional Optical Microscopic Images of Solid Plate and Porous Foam Electrodes after Electrolysis

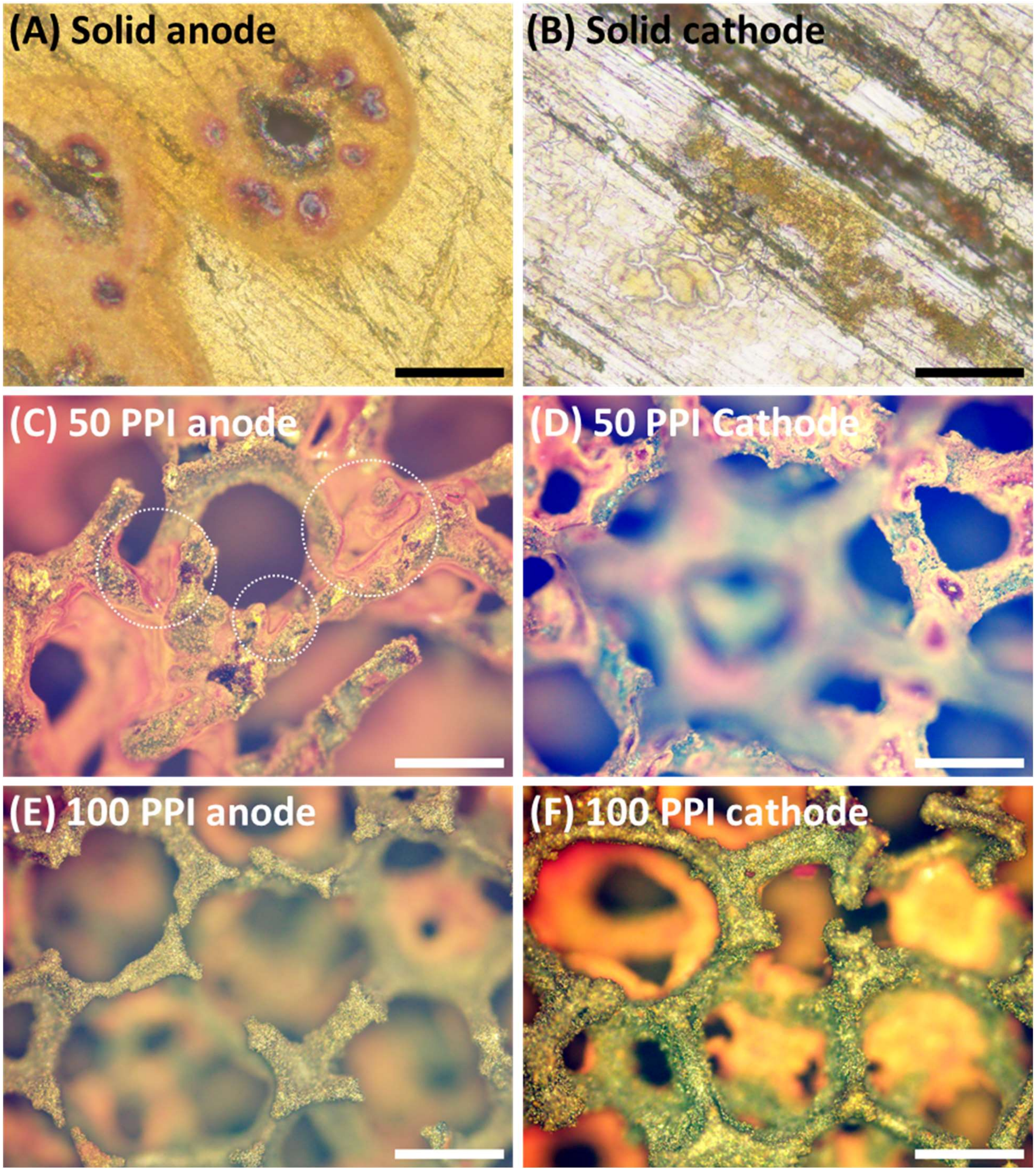

Figure S3. (A) Solid plate anode after electrolysis illustrating pits (scale: 50 μm), (B) Solid plate cathode after electrolysis with light brown-colored deposits (scale: 50 μm), (C) Pits growing in irregular directions found on 50 PPI anode (white circles, scale: 50 μm), (D) Insignificant pore clogging in 50 PPI cathode (scale:

50 µm), (E) Relatively clean outer-layer pore structures of 100 PPI porous anode (scale: 500 µm) and (F) the similar feature of 100 PPI cathode (scale: 500 µm).

Section S6. Optical Microscopy Images of Porous Foam Cathodes Indicative of Chemical Dissolution

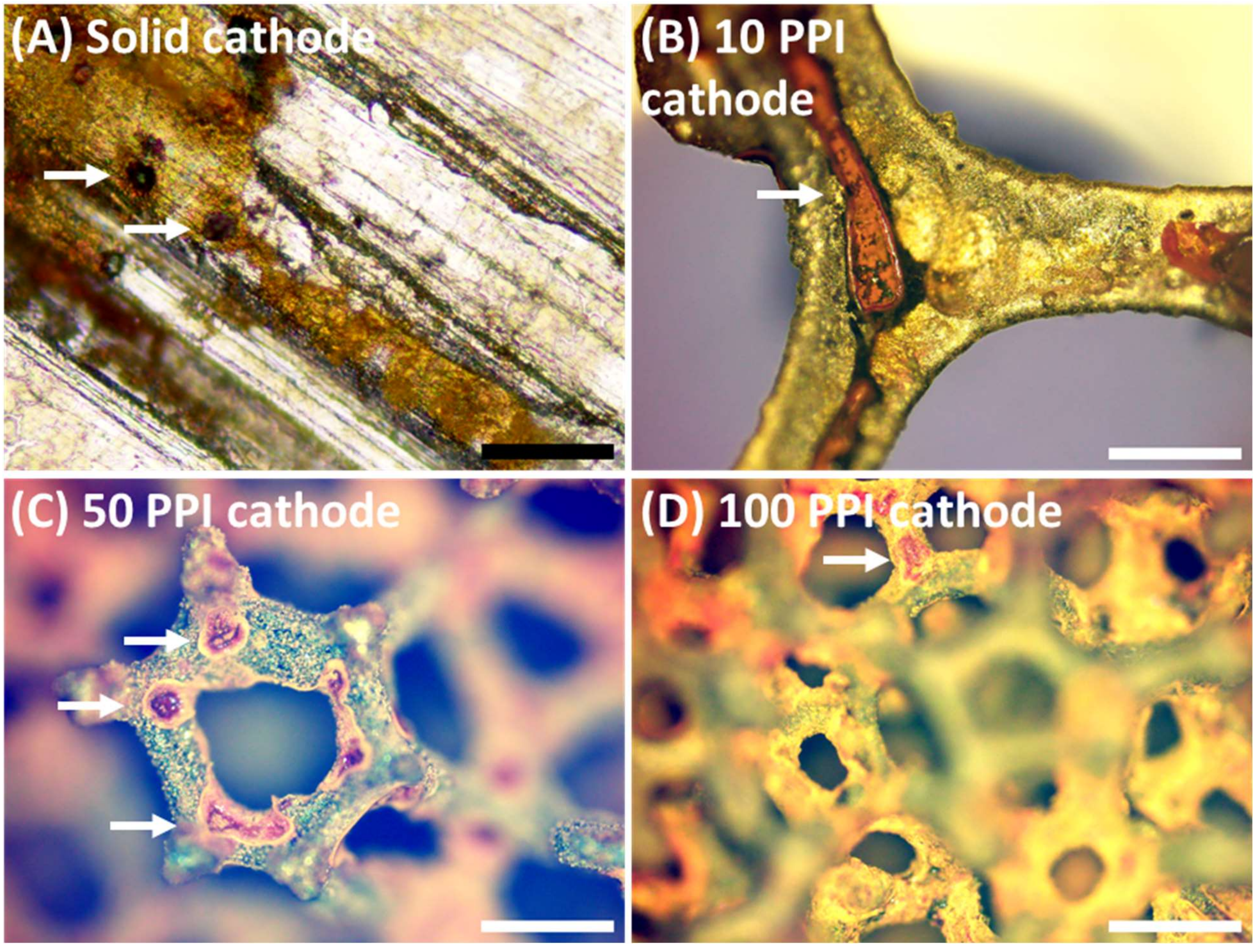

Figure S4. Images of solid and porous cathode indicative of chemical dissolution. (A) Solid plate cathode (scale: 50 μm), (B) 10 PPI cathode (scale: 500 μm), (C) 50 PPI cathode (scale: 500 μm), and (D) 100 PPI cathode (scale: 500 μm).

Section S7. Quantitative Analysis of Porous Cylindrical Electrodes using nCT

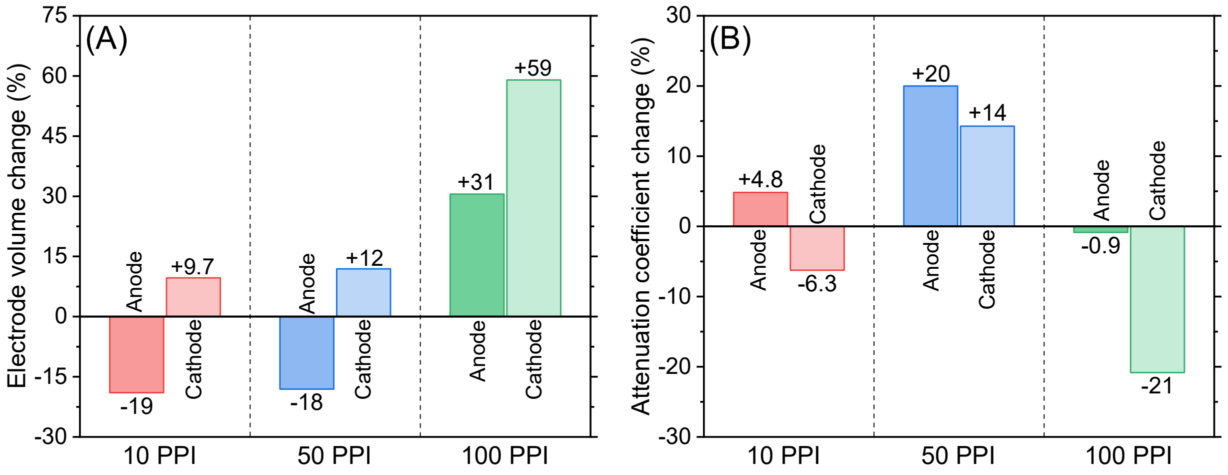

Figure S5. Changes in the physicochemical properties of porous anode and cathode before and after 40 min of electrolysis estimated by neutron tomography. (A) Overall electrode volume change. (B) Overall attenuation coefficient change.

Quantitative data representing the entire electrode were extracted from the tomograms. The relative total volume change after electrolysis was calculated with respect to the unused electrode (Figure S5A). The total volume change of the 10 PPI anode was found to be -19% (i.e., volume reduction by 19%) as expected given the sacrificial nature of the anode. The optical image is shown in Figure 4B2 in the manuscript where irregular pits were occasionally seen, while surface deposition was insignificant. About 10% volume increase of the 10 PPI cathode also coincided with the visual observation of severe surface coverage observed in Figure 4B3 in the manuscript. These observations were also in agreement with the mass change of the electrodes (Figure S6). Similar trends were found with the 50 PPI electrodes. The 50 PPI anode lost 18% of its volume, indicating a dominant impact of mass loss during the electrolysis over the patchy growth of corrosion layers found by optical microscopy. The cathode volume increased by 12%, which was somewhat unexpected given the only moderate surface deposition observed in Figure 4C3 in the manuscript, which suggests that internal structures (not observable using the optical microscope) might suffer more severe precipitate deposition. Interestingly, the 100 PPI anode and cathode showed a substantial volume increase by 31 and 59%, respectively, well-matching the optical

microscopic observation of the exclusive pore-clogging deposits. Moreover, it should be noted that, unlike the other two cases of 10 and 50 PPI anodes, mass decline despite the volume increase was observed with the 100 PPI anode (Figure S6), suggesting a highly porous nature of iron precipitates (Figure S7).

The overall attenuation coefficient change was indicative of chemical alterations, not only by ferric oxyhydroxide but also by the deposition of other elements (Figure S5B). A slight increase in the attenuation coefficient of the 10 PPI anode was attributed to H in ferric oxyhydroxide overwhelming the low neutron attenuating O, while the opposite result of the cathode hinted the dominating effect of deposited elements with low attenuating coefficients on the overall value, counter-intuitive to the visual observation shown in Figure 4B3 in the manuscript. Based on the previous studies on $CaCO_3$ and $Mg(OH)_2$ precipitate formation on the cathodic surface due to the locally basic pH environment[5-7], a decrease in the overall coefficient of the cathode was attributed to C, O, Ca, and Mg whose attenuation coefficients are lower by several orders of magnitude than those of Fe and H[8]. A substantial increase in the coefficients of 50 PPI anode and cathode pronounced the deposition of hydrogenated substances, probably ferric oxyhydroxide, dominating other low-attenuating elements like Ca, Mg, and Si, especially on the cathode (Figure 8 in the manuscript). 100 PPI anode showed only marginal change in the overall coefficient, which was rationalized by the concurrent accumulation of low-neutron-attenuating elements such as Si compensating coefficient increase by H in ferric hydroxide (Figure 8 in the manuscript). A substantial decline of attenuation coefficient, particularly for the cathode, by 21% suggested a dominating impact of $CaCO_3$ and $Mg(OH)_2$ deposition that was not visually identifiable (Figure 4D3 in the manuscript).

Section S8. Cylindrical Porous Electrode Mass Change

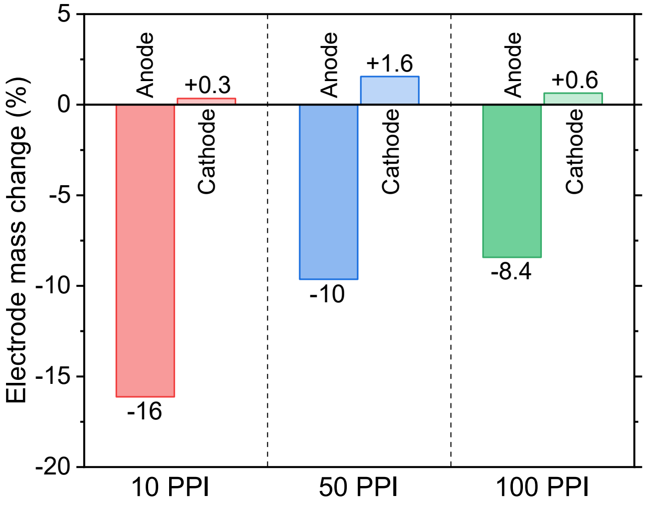

Figure S6. Relative change in porous electrode mass induced by 40 min electrocoagulation.

Section S9. SEM of cylindrical 100 PPI anode before and after EC.

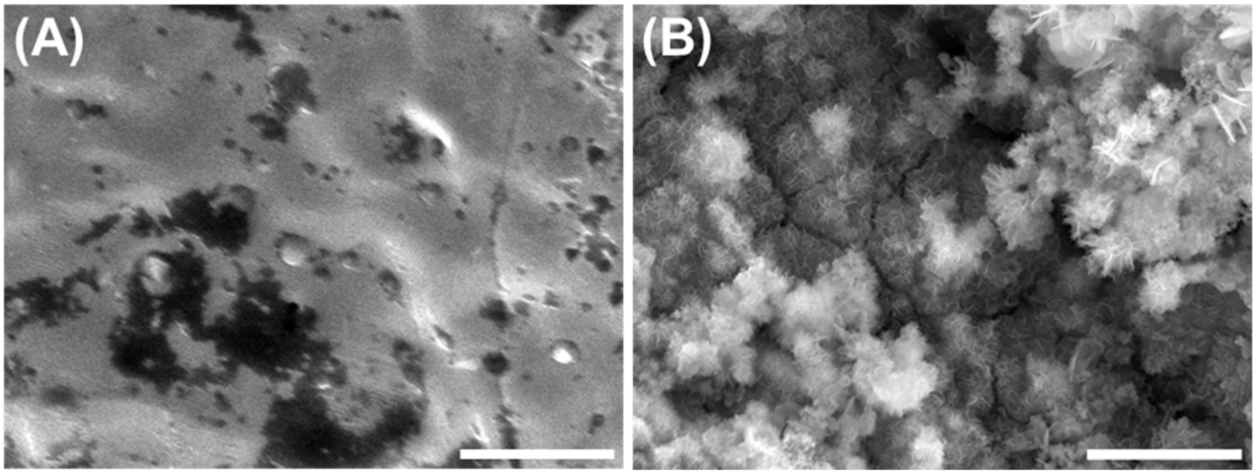

Figure S7. Highly porous nature of surface deposits found on cylindrical 100 PPI anode after 40 minutes of electrocoagulation operation; Before (A) and after (B) electrocoagulation. Scale bars represent 5 μm.

Section S10. Relative Changes of Electrode Volume and Attenuation Coefficient Estimated by Neutron Tomograms of Cylindrical Porous Electrodes

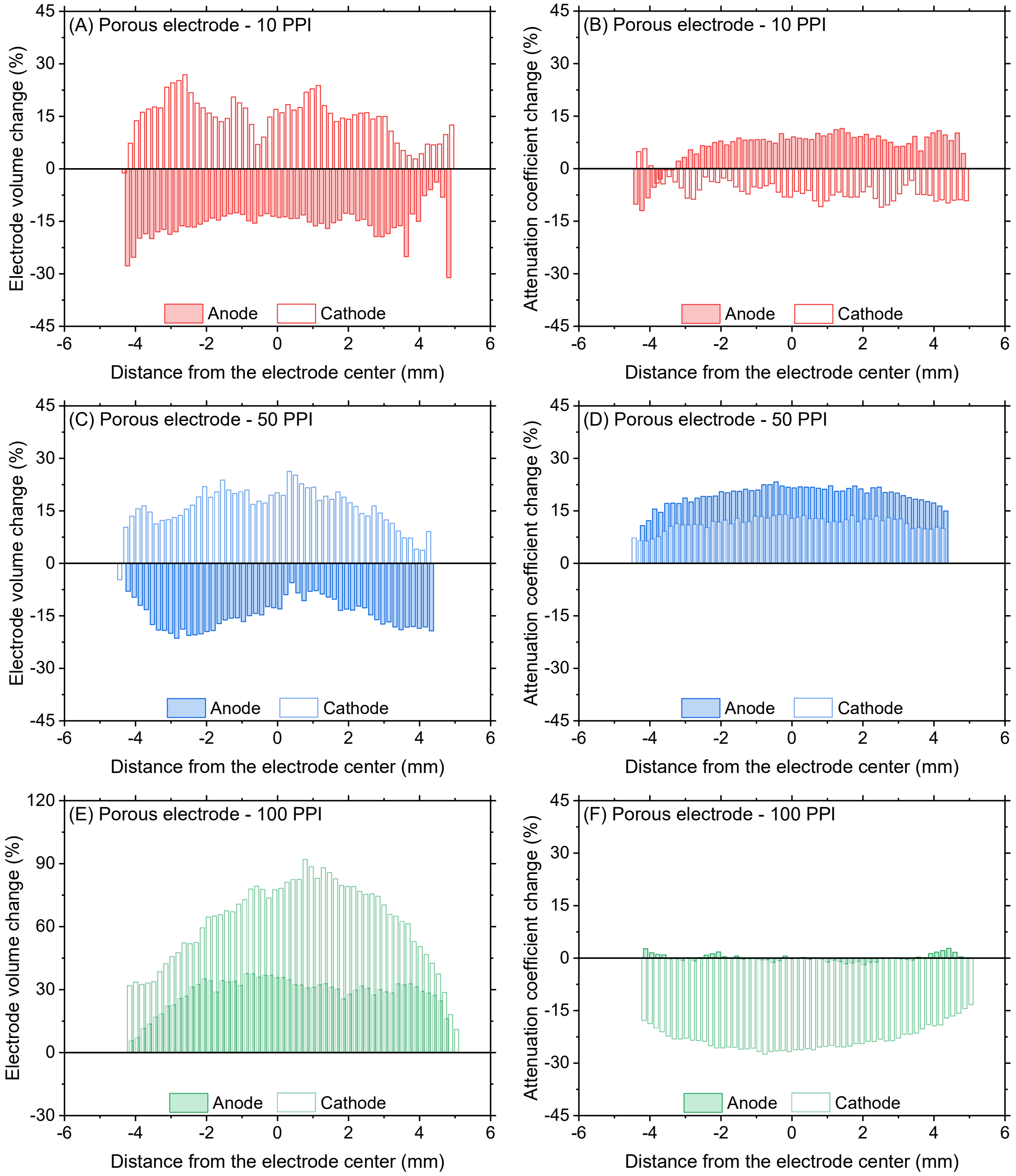

Figure S8. Relative changes of electrode volume and neutron attenuation coefficient before and after electrocoagulation along the electrode thickness.

Section S11. Electrical Energy Consumption Comparison between Solid and Porous Electrodes in a Flow-through Electrocoagulation System

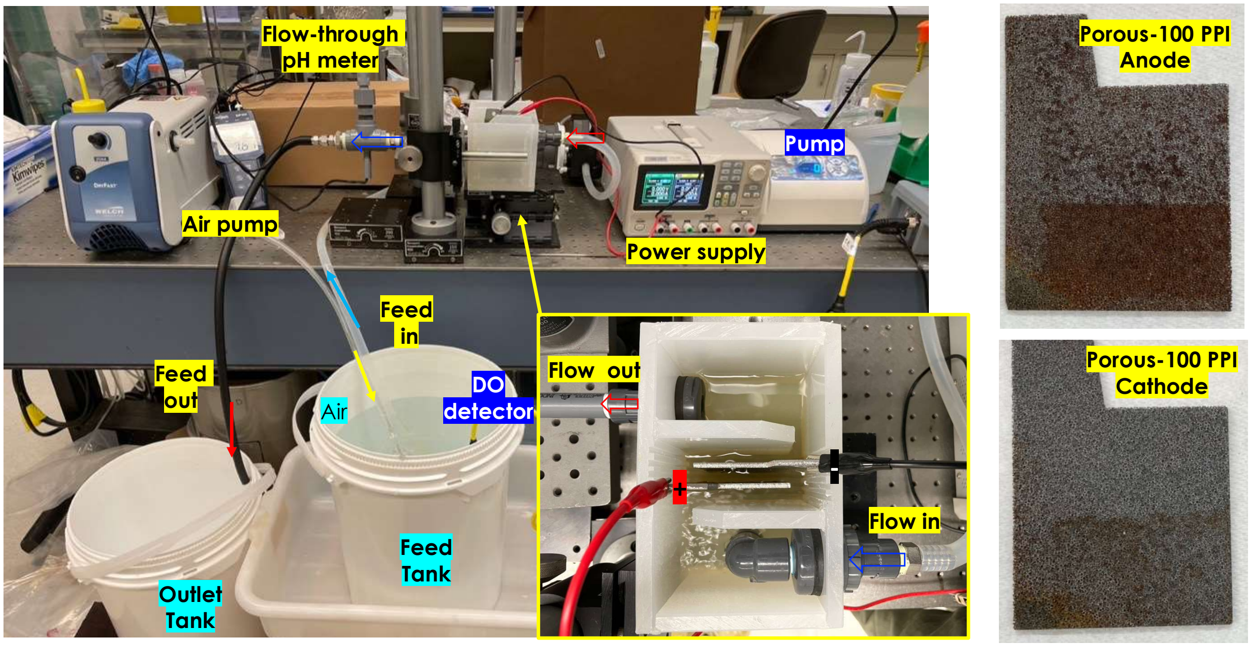

Figure S9. Continuous flow-through electrocoagulation operation. The yellow-boxed inset depicts details of the reactor wherein electrodes were placed. The photo on the right shows the iron foam electrodes after EC with 0.5 L/min of flowrate at 10 mA/cm^2**.**

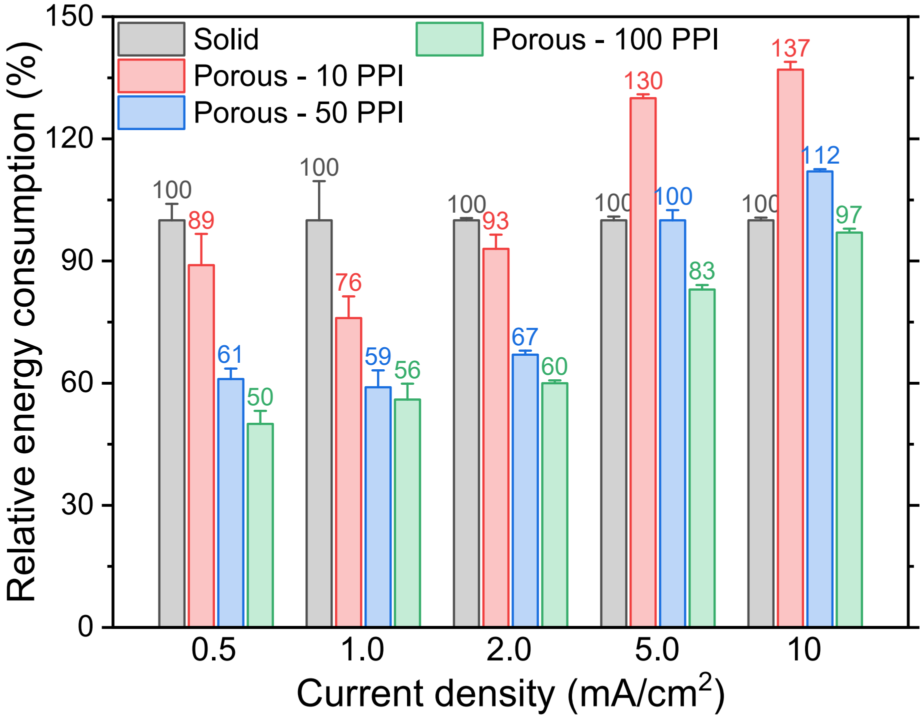

Figure S10. Relative electrical energy consumption with porous electrodes at different current densities with respect to the solid electrode when electrocoagulation was performed in a batch operation ‘in a flow-through reactor system’. Averages and standard deviations are represented.

References

(1) Abada, B.; Joag, S.; Alspach, B.; Bustamante, A.; Chellam, S. Inorganic and Organic Silicon Fouling of Nanofiltration Membranes during Pilot-Scale Direct Potable Reuse. *ACS ES&T Engineering* **2023**, *3* (9), 1413-1423. DOI: 10.1021/acsestengg.3c00172.

(2) Abada, B.; Safarik, J.; Ishida, K. P.; Chellam, S. Surface characterization of end-of-life reverse osmosis membranes from a full-scale advanced water reuse facility: Combined role of bioorganic materials and silicon on chemically irreversible fouling. *Journal of Membrane Science* **2022**, *653*, 120511. DOI: 10.1016/j.memsci.2022.120511.

(3) Abada, B.; Safarik, J.; Ishida, K. P.; Chellam, S. Elucidating Foulant Diversity during Full-Scale Potable Reuse: Forensic Analysis of Lead and Lag Elements of a Three-Stage Reverse Osmosis System. *ACS ES&T Engineering* **2022**, *2* (11), 2116-2129. DOI: 10.1021/acsestengg.2c00171.

(4) Lakshmanan, D.; Clifford, D. A.; Samanta, G. Ferrous and ferric ion generation during iron electrocoagulation. *Environmental Science & Technology* **2009**, *43* (10), 3853-3859. DOI: 10.1021/es8036669.

(5) Müller, S.; Behrends, T.; van Genuchten, C. M. Sustaining efficient production of aqueous iron during repeated operation of Fe(0)-electrocoagulation. *Water Research* **2019**, *155*, 455-464. DOI: 10.1016/j.watres.2018.11.060.

(6) Yang, Q.; Xu, L.; He, Q.; Wu, D. Reduced cathodic scale and enhanced electrochemical precipitation of Ca^{2+} and Mg^{2+} by a novel fenced cathode structure: Formation of strong alkaline microenvironment and favorable crystallization. *Water Research* **2021**, *209*, 117893. DOI: 10.1016/j.watres.2021.117893.

(7) James, M.; Mitch, W. A. Electrochemical generation of hydroxide for precipitative water softening. *ACS ES&T Water* **2022**, *2* (12), 2677-2685. DOI: 10.1021/acsestwater.2c00451.

(8) Bacon, G. E.; Lonsdale, K. Neutron diffraction. *Reports on Progress in Physics* **1953**, *16* (1), 1. DOI: 10.1088/0034-4885/16/1/301.
